# Performance of distinct microbial based solutions in a Campylobacter infection challenge model in poultry

**DOI:** 10.1186/s42523-021-00157-6

**Published:** 2022-01-03

**Authors:** Maxine Ty, Khaled Taha-Abdelaziz, Vanessa Demey, Mathieu Castex, Shayan Sharif, John Parkinson

**Affiliations:** 1 Department of Biochemistry, University of Toronto, Toronto, ON Canada; 2 Program in Molecular Medicine, Hospital for Sick Children, Peter Gilgan Center for Research and Learning, 686 Bay Street, Toronto, ON M5G 0A4 Canada; 3 Animal and Veterinary Sciences Department, Clemson University, Clemson, SC 29634 USA; 4 Lallemand SAS, Blagnac, France; 5 Department of Pathobiology, Ontario Veterinary College, University of Guelph, Guelph, ON N1G 2W Canada; 6 Department of Molecular Genetics, University of Toronto, Toronto, ON Canada

**Keywords:** Probiotics, *Campylobacter jejuni*, Microbiome, Infection challenge

## Abstract

**Background:**

Antibiotic growth promoters (AGPs) are commonly used within poultry production to improve feed conversion, bird growth, and reduce morbidity and mortality from clinical and subclinical diseases. Due to the association between AGP usage and rising antimicrobial resistance, the industry has explored new strategies including the use of probiotics and other microbial-based interventions to promote the development of a healthy microbiome in birds and mitigate against infections associated with food safety and food security. While previous studies have largely focused on the ability of probiotics to protect against *Clostridium perfringens* and *Salmonella enterica*, much less is known concerning their impact on *Campylobacter jejuni*, a near commensal of the chicken gut microbiome that nevertheless is a major cause of food poisoning in humans.

**Results:**

Here we compare the efficacy of four microbial interventions (two single strain probiotics, the bacterium—*Pediococcus acidilactici*, and the yeast—*Saccharomyces cerevisiae boulardii*; and two complex, competitive exclusion, consortia—Aviguard and CEL) to bacitracin, a commonly used AGP, to modulate chicken gut microbiota and subsequently impact *C. jejuni* infection in poultry. Cecal samples were harvested at 30- and 39-days post hatch to assess *Campylobacter* burden and examine their impact on the gut microbiota. While the different treatments did not significantly decrease *C. jejuni* burden relative to the untreated controls, both complex consortia resulted in significant decreases relative to treatment with bacitracin. Analysis of 16S rDNA profiles revealed a distinct microbial signature associated with each microbial intervention. For example, treatment with Aviguard and CEL increased the relative abundance of *Bacteroidaceae* and *Rikenellaceae* respectively. Furthermore, Aviguard promoted a less complex microbial community compared to other treatments.

**Conclusions:**

Depending upon the individual needs of the producer, our results illustrate the potential of each microbial interventions to serve flock-specific requirements.

**Supplementary Information:**

The online version contains supplementary material available at 10.1186/s42523-021-00157-6.

## Introduction

Subtherapeutic doses of antimicrobial growth promoters (AGPs) are commonly used within poultry production to supplement bird diets to improve feed conversion, bird growth, and reduce morbidity and mortality from clinical and subclinical diseases [1]. Since AGPs are not absorbed from the intestines, they do not act therapeutically and have no systematic effects [1]. While the exact mechanism by which AGPs promote growth remains unknown, it has been proposed that they operate through modulating the gut microbiota [2]. One hypothesis is that AGPs exert an overall antibacterial effect, which may protect nutrients from bacterial destruction, decrease production of toxins by intestinal bacteria, and/or reduce incidence of subclinical intestinal infection [1]. Consequently, AGPs have been seen as offering prophylactic benefits by mitigating against infectious agents such as *Clostridium perfringens*, the causative agent of necrotic enteritis, capable of decimating entire flocks [3–5]. Although AGPs have been shown to be effective against *C. perfringens*, their impact against other pathogens, particularly those associated with food safety such as *Campylobacter jejuni* is less documented [4, 6–8]. *C. jejuni* is considered to possesses a near commensal relationship with chickens and is typically acquired after three weeks of age resulting in asymptomatic infections [9, 10]. In contrast, in humans *C. jejuni* is a leading source of foodborne illness in industrialized countries [11]. With links between the use of AGPs and the ability of these pathogens to acquire novel mechanisms of antimicrobial resistance there is an urgent need to identify efficacious alternatives, capable of promoting the development of a healthy microbiome [1, 3].

To address global bans on the use of AGPs, the poultry industry has explored the use of probiotics, natural microbes that confer a beneficial effect on their host [12, 13]. Single strain probiotic species including species of *Streptococcus*, *Bacillus*, *Bifidobacterium*, *Enterococcus*, *Lactobacillus, Pediococcus* and *Saccharomyces*, have been shown to have beneficial effects on broiler performance, laying hens, modulation of intestinal gut microbiota and pathogen inhibition [13–16]. However, probiotics have shown varying results between products and farms [17], limiting our understanding of their specific impact on the composition of the gut microbiome. In the case of probiotics this may be in part explained by recent findings of person-, region- and strain-specific patterns of sensitivity to colonization by probiotics in humans [18]. In attempts to overcome these limitations, the use of more complex communities that engraft within the gastro-intestinal (GI) tract, stabilized through mutualistic interactions that rely on metabolic cooperation to exploit specific substrates, is receiving renewed attention [19, 20]. Such interest has been driven by recent successes of fecal microbiota transplantation in humans (FMT) [21, 22]. Applied to poultry, FMTs have been shown to increase feed efficiency, growth performance, immune function and to reduce bacterial infections such as *Salmonella* [23, 24]. In challenge experiments, transfer of a whole mature microbiome to newly-hatched chicks also reduced transmission and colonisation of *C. jejuni* [25], reduced mortality from necrotizing enteritis (NE) [26] and decreased colonization by pathogenic and antibiotic resistant *E. coli* [27].

Given the range of microbial based products available and limited data on their impact on *C. jejuni* infections, we sought to compare a set of four industrially-relevant microbial interventions to bacitracin, an AGP that has been widely used, on their efficacy against a model of *C. jejuni* infection in poultry [28]. Among these products were two single strain probiotics: *Pediococcus acidilactici* and *Saccharomyces boulardii*; a commercial competition exclusion product, Aviguard; and another experimental competition exclusion, CEL. In addition, we performed 16S rRNA sequence surveys to explore the ability of each product to engraft within the GI tract, alter the composition of the GI microbiome and identify taxa that might be associated with reduction of *C. jejuni* burden. We further investigated the impact of *C. jejuni* on gut microbiome in the presence and absence of AGPs.

## Results

### Two of four microbial solutions reduced colonization of *C. jejuni* in mature broilers relative to bacitracin

**Fig. 1:**
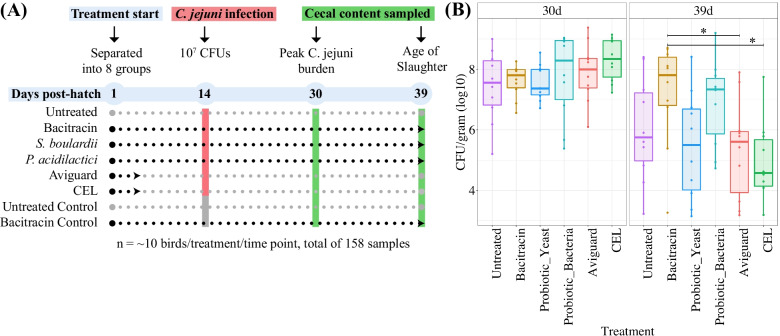
Schematic of Experimental Design and *C. jejuni* burden. **A** Experimental design. Chickens were divided randomly into 8 groups and assigned to either one of 6 treatments or left untreated at day one of age (Aviguard, CEL, probiotic bacteria (*Pediococcus acidilactici CNCMI-4622*), probiotic yeast (*Saccharomyces cerevisiae boulardii CNCMI-1079*), AGP (bacitracin)). On day 14, all groups, except for two (one bacitracin and one untreated group) were orally challenged with 10^7^ CFUs of *C. jejuni* (strain 81–176). Samples of cecal contents were collected at day 30 and 39 of age (n = 10 birds per time point and treatment group, total of 158 samples for CFU enumeration and 16S rRNA sequencing). After sample collection, tenfold serial dilutions in PBS were plated onto Muller Himton agar containing Preston *Campylobacter* Selective Supplement. Plates were incubated in microaerophilic conditions at 41 °C and CFUs of *C. jejuni* were enumerated after 40–48 h and expressed as log_10_ *Campylobacter* /g of cecal content. **B** Boxplots representing the median of CFUs of *C. jejuni* in each treatment group. Statistical analysis was performed using Kruskal–Wallis test followed by pairwise Dunn's tests. *P < 0.05

The purpose of this study was to monitor the impact of select microbial solutions on the chicken gut microbiome and their ability to reduce colonization by *C. jejuni*. To that end, we performed a challenge trial involving a total of 160 broiler chickens (Ross 708; Fig. 1A). One day old chicks were split into 8 groups and subjected to different treatments (see Methods). Four groups received one of the following products: *Pediococcus acidilactici* (a probiotic bacteria)*; Saccharomyces cerevisiae boulardii* (a probiotic yeast); Aviguard (a commercially available probiotic consortium); and CEL (an experimental probiotic consortium). Two groups received bacitracin (AGP) and two groups were left untreated. At day 14, all groups were orally challenged with *C. jejuni* apart from two groups to act as control (one bacitracin and one untreated group). At days 30 and 39 post hatch (timepoints associated with peak *C. jejuni* colonization and broiler slaughter), birds were sacrificed and cecal contents recovered for 16S rDNA sequencing.

Based on cecal colony forming units (CFUs), we found that at 30 days post-hatch none of the probiotic products or bacitracin resulted in significant differences in *C. jejuni* burden relative to the untreated control (Fig. 1B). At 39 days post hatch, we found that treatment with Aviguard and CEL exhibited a significant decrease in *C. jejuni* burden relative to birds treated with bacitracin (*P* < 0.05, Post-hoc pairwise Dunn’s Test). While *S. cerevisiae boulardii* resulted in a notable decrease in *C. jejuni* burden relative to bacitracin, this decrease was not found to be statistically significant. We observed no significant differences between any of these treatments and the untreated control group. Aviguard’s ability to lower *C. jejuni* burden in our study mirrors the success found in other studies where it was able to inhibit intestinal colonization of *Clostridium perfringens* and *Salmonella typhimurium* in chickens [29–32]. As such, these results suggest replacing dietary bacitracin with Aviguard, in addition to protecting against necrotic enteritis induced by *C. perfringens*, as well as colonization by *S. typhimurium*, may additionally result in a significant reduction in *C. jejuni* burden.

### Alpha diversity in the cecal microbial community increases with age, *C. jejuni* challenge, and bacitracin AGP treatment

Given the decrease in *C. jejuni* burden associated with the microbial consortia, we were interested in understanding how the cecal microbiota responds to each treatment and challenge. From the cecal samples harvested from each bird at day 30 and day 39 post hatch, we extracted DNA and performed 16S rDNA surveys. Overall, 4,519,255 paired-end reads were generated. After quality filtering, we retained 2,195,510 paired-end reads with a median of 13,562 reads (5,753–29,542 reads; Additional file 12: Figure S1A) per sample. Sequences were clustered into 1,305 Operational Taxonomic Units (OTUs) based on a de novo assignment with similarity set at 97% (Additional file 12: Figure S1B). Of these, 17 OTUs, represented by 75 reads (0.003% of retained reads) assigned as bacteria, could not be assigned to a phylum. A further 259 OTUs (represented by 72,748 or 3.3% of reads) could not be assigned to a family and 928 OTUs (represented by 961,941 or 43.8% of reads) could not be assigned to a genus. Across all samples, our results confirmed *Firmicutes* as the major phyla in the adult chicken gut microbiome, represented by 89% ± 14% (mean ± std dev) of assigned reads, compared to *Bacteroidetes* at 7.1% ± 13%. The most abundant families within the *Firmicutes* phylum were *Ruminococcaceae* represented by 34% ± 11% of assigned reads, followed by *Clostridiales vadinBB60 group* at 24% ± 12% and *Lachnospiraceae* at 24% ± 11%. The *Bacteroidetes* phylum consisted of *Bacteroidaceae* at 3.6% ± 11% and *Rikenellaceae* at 3.5% ± 8.4%. *C. jejuni* a member of *Epsilonproteobacteria* was represented by 0.14% ± 0.30% of all assigned reads. *C. jejuni* sequence counts exhibited significant correlation with the CFUs of *C. jejuni* obtained above at both time points (p < 0.001; Spearman’s rank correlation coefficient; Additional file 12: Figure S2A and B). Consistent with the negative CFU results reported for the two unchallenged control groups, we did not find any reads mapping to *C. jejuni* in either of these samples (Additional file 13: Figure S3). Given that 16S rDNA data provide only information on *relative* and not *absolute* abundance, we rely on CFUs as a more reliable indicator of *C. jejuni* burden to compare treatment effect on pathogen clearance. Furthermore, since only the 39-day samples yielded significant differences in *C. jejuni* burden, we focus subsequent analyses on samples associated with this later timepoint.

**Fig. 2:**
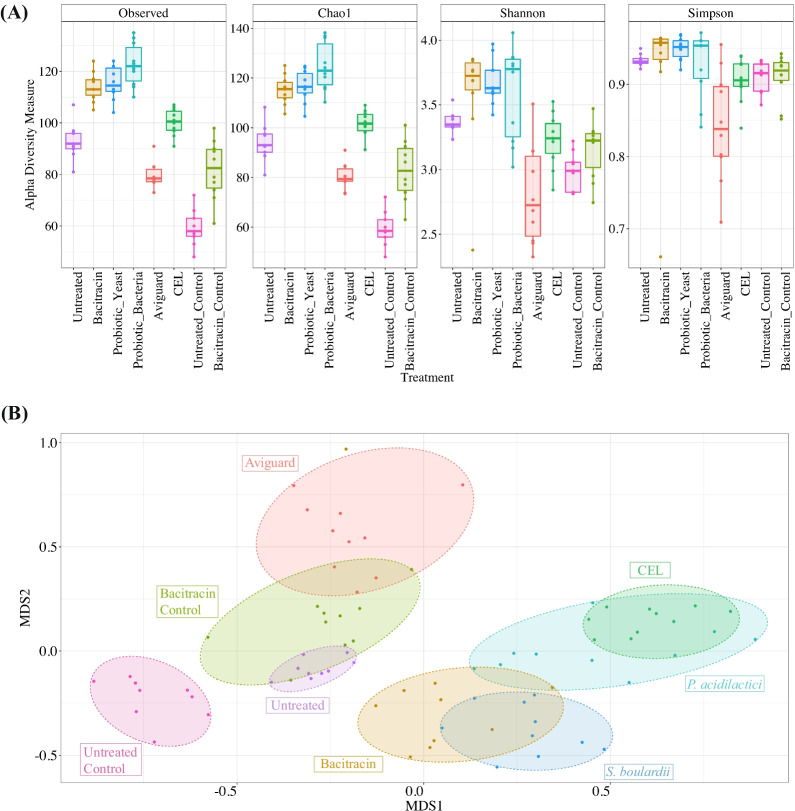
Alpha and beta diversity of cecal microbial communities. **A** Boxplots representing alpha diversity metrics of richness (observed number of OTUs and Chao1) and evenness (Shannon and Simpson) for 39-day samples grouped according to treatment (no. of OTUs at 97% similarity). Each point represents the diversity score for a sample, colour-coded according to treatment. **B** Non-metric multidimensional scaling (nMDS) plot based on Bray–Curtis dissimilarity matrix on relative abundance data in 39-day samples. Colours indicate treatment group

Using four measures of alpha diversity, two measures of species richness (observed number of OTUs and Chao1) and two measures of species evenness (Shannon and Simpson indices), we identified a spectrum of diversity across treatments at day 39 (Fig. 2A). Across all treatment groups, the unchallenged and untreated exhibited the lowest species richness, while the *P. acidilactici* group had the highest (post-hoc pairwise Wilcoxon of Observed and Chao1; Table 1; Fig. 2A). The former finding potentially highlights the lack of any influence from either treatment and/or challenge with *C. jejuni*. Samples from birds treated with *P. acidilactici* also exhibited the highest species evenness (post-hoc pairwise Wilcoxon of Shannon and Simpson; Table 1; Fig. 2A). Interestingly, we found that Aviguard samples, which exhibited the lowest species richness across the challenged groups, were also associated with notably lower species evenness, suggesting the promotion of a relatively less complex microbiome that is less evenly distributed relative to the other treatments (post-hoc pairwise Wilcoxon of Observed, Chao1, Shannon and Simpson; Table 1; Fig. 2A).

**Table 1:** Significant differences in alpha diversity indices in pairwise comparisons between treatments

	Aviguard	Bacitracin	Bacitracin_Control	CEL	Probiotic_Bacteria	Probiotic_Yeast	Untreated
**30-day samples**							
Observed							
Bacitracin	**						
Bacitracin_Control	–	*					
CEL	**	–	**				
Probiotic_Bacteria	***	**	***	**			
Probiotic_Yeast	**	–	**	–	***		
Untreated	**	–	*	–	***	–	
Untreated_Control	**	**	**	***	***	***	***
Chao1							
Bacitracin	*						
Bacitracin_Control	–	*					
CEL	**	–	**				
Probiotic_Bacteria	***	**	***	***			
Probiotic_Yeast	*	–	**	–	***		
Untreated	*	–	*	*	***	–	
Untreated_Control	**	***	**	***	***	***	***
Shannon							
Bacitracin	**						
Bacitracin_Control	***	–					
CEL	***	–	–				
Probiotic_Bacteria	***	***	***	***			
Probiotic_Yeast	***	–	–	–	**		
Untreated	***	–	**	–	**	–	
Untreated_Control	***	–	–	–	***	–	**
Simpson							
Bacitracin	**						
Bacitracin_Control	***	–					
CEL	***	–	–				
Probiotic_Bacteria	***	**	**	**			
Probiotic_Yeast	***	–	–	–	*		
Untreated	***	–	*	*	**	–	
Untreated_Control	**	–	–	–	**	–	–
**39-day samples**							
Observed							
Bacitracin	***						
Bacitracin_Control	–	***					
CEL	***	***	***				
Probiotic_Bacteria	***	*	***	***			
Probiotic_Yeast	***	–	***	***	–		
Untreated	**	***	*	*	***	***	
Untreated_Control	***	***	***	***	***	***	***
Chao1							
Bacitracin	***						
Bacitracin_Control	–	***					
CEL	***	***	**				
Probiotic_Bacteria	***	*	***	***			
Probiotic_Yeast	***	–	***	***	–		
Untreated	***	***	*	*	***	***	
Untreated_Control	***	***	**	***	***	***	***
Shannon							
Bacitracin	**						
Bacitracin_Control	*	**					
CEL	*	**	–				
Probiotic_Bacteria	**	–	*	–			
Probiotic_Yeast	***	–	***	***	–		
Untreated	**	*	**	-	–	***	
Untreated_Control	–	**	–	*	**	***	***
Simpson							
Bacitracin	*						
Bacitracin_Control	–	*					
CEL	–	*	–				
Probiotic_Bacteria	*	–	–	–			
Probiotic_Yeast	**	–	**	**	–		
Untreated	*	–	–	*	–	*	
Untreated_Control	–	*	–	–	–	**	*

*P*-values were calculated using post-hoc pairwise Wilcoxon rank sum tests for four indices (Observed, Chao1, Shannon, Simpson). Samples are grouped by time point

*p < 0.05; **p < 0.01; ***p < 0.001

Comparing the two unchallenged control groups (untreated and bacitracin) with their respective challenged groups, we found that *C. jejuni* challenge resulted in increased species richness and increased species evenness at 39 days (post-hoc pairwise Wilcoxon of Observed, Chao1, Shannon and Simpson; Table 1; Fig. 2A). Comparing 39-day samples between the bacitracin and untreated group, we also found that bacitracin treatment increased species richness, in the absence and presence of *C. jejuni* (post-hoc pairwise Wilcoxon of Observed and Chao1; Table 1; Fig. 2A). In contrast, bacitracin treatment had no impact on species evenness relative to the untreated group.

### Each treatment results in a unique microbial signature

We were next interested in comparing the impact of each treatment on the makeup of the microbial communities in the ceca. Using the Bray–Curtis metric as a measure of beta-diversity, we conducted permutational multivariate analysis of variance (PERMANOVA) and permutational analysis of multivariate dispersions (PERMDISP) to measure whether there is a significant separation of samples by treatment or time point due to differences in microbiome structure. PERMANOVA evaluates the significance of separation between centroids of samples grouped by treatment or time point but is sensitive to multivariate dispersion. PERMDISP assesses if the distribution or spread of two sample groups are significantly different and is conducted in parallel to PERMANOVA. For example, a non-significant PERMDISP result would reveal that two sample groups have similar dispersions, which places confidence in a significant PERMANOVA result as it is likely not due to differences in group dispersions. A significant PERMDISP result would mean that two sample groups have different dispersions, therefore a significant PERMANOVA result cannot be interpreted to sample groups having significant separation [33]. To assess if different treatment groups were significantly associated with microbial community structure, we conducted pairwise PERMANOVA and PERMDISP on samples grouped by time point and treatment. Results from these analyses showed that, although most treatment groups at 39 days had significant separation from each other, untreated birds challenged with *C. jejuni* did not exhibit a significantly different community structure from challenged birds treated with bacitracin, *P. acidilactici* or *S. cerevisiae boulardii* (Table 2; Fig. 2B). Consistent with the comparisons of alpha diversity, *C. jejuni* challenge resulted in a significantly different community structure in both the untreated and bacitracin groups (Table 2; Fig. 2B).

**Table 2:** Significant differences in microbial community structure in pairwise comparisons between treatments

	Aviguard	Bacitracin	Bacitracin_Control	CEL	Probiotic_Bacteria	Probiotic_Yeast	Untreated
**30-day samples**							
PERMANOVA							
Bacitracin	**						
Bacitracin_Control	**	**					
CEL	**	**	**				
Probiotic_Bacteria	**	**	**	**			
Probiotic_Yeast	**	**	**	**	**		
Untreated	**	**	**	**	**	**	
Untreated_Control	**	**	**	**	**	**	**
PERMDISP							
Bacitracin	–						
Bacitracin_Control	–	–					
CEL	–	–	–				
Probiotic_Bacteria	–	–	–	–			
Probiotic_Yeast	–	–	–	–	–		
Untreated	–	–	–	–	–	–	
Untreated_Control	–	–	–	–	–	–	–
PERMANOVA							
39-day samples							
Bacitracin	**						
Bacitracin_Control	**	**					
CEL	**	**	**				
Probiotic_Bacteria	**	**	**	**			
Probiotic_Yeast	**	**	**	**	**		
Untreated	**	**	**	**	**	**	
Untreated_Control	**	**	**	**	**	**	**
PERMDISP							
Bacitracin	–						
Bacitracin_Control	–	–					
CEL	–	–	–				
Probiotic_Bacteria	–	–	–	–			
Probiotic_Yeast	–	–	–	–	–		
Untreated	–	*	–	–	*	*	
Untreated_Control	–	–	–	–	–	–	–

*P* values measured by pairwise PERMANOVA and PERMDISP tests of the Bray–Curtis distances conducted in parallel

*p < 0.05; **p < 0.01; ***p < 0.001

**Fig. 3:**
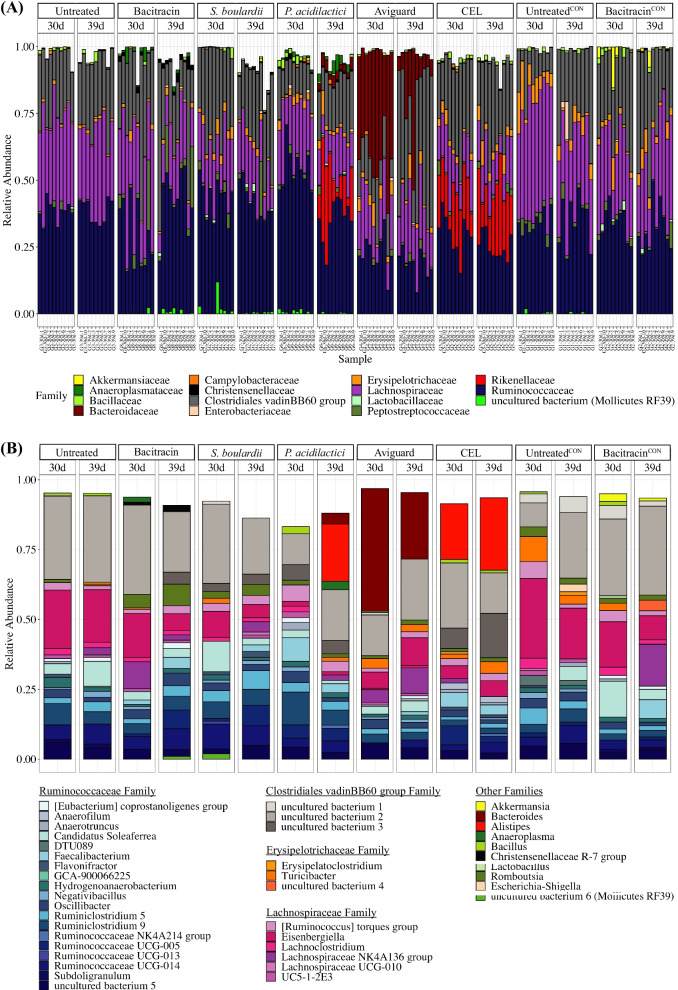
Microbial community composition of chicken cecal content. **A** Stacked bar plots representing relative abundances of the top 15 families in all samples, grouped by treatment and age. Legend lists taxonomic families in order of appearance **B** Stacked bar plots representing relative abundances of the top 20 genera in each treatment and timepoint

Consistent with observations from the beta diversity analyses, assigning the 1305 OTUs to discrete taxa revealed unique microbial profiles for each treatment and time point (Fig. 3A, B). For example, at both timepoints, treatment with CEL (and *P. acidilactici* at 39 days), resulted in relatively high abundances of *Rikenellaceae*; in contrast Aviguard resulted in a high abundance of *Bacteroidaceae*; while bacitracin yielded signatures associated with *Akkermansiaceae* among unchallenged birds.

**Fig. 4:**
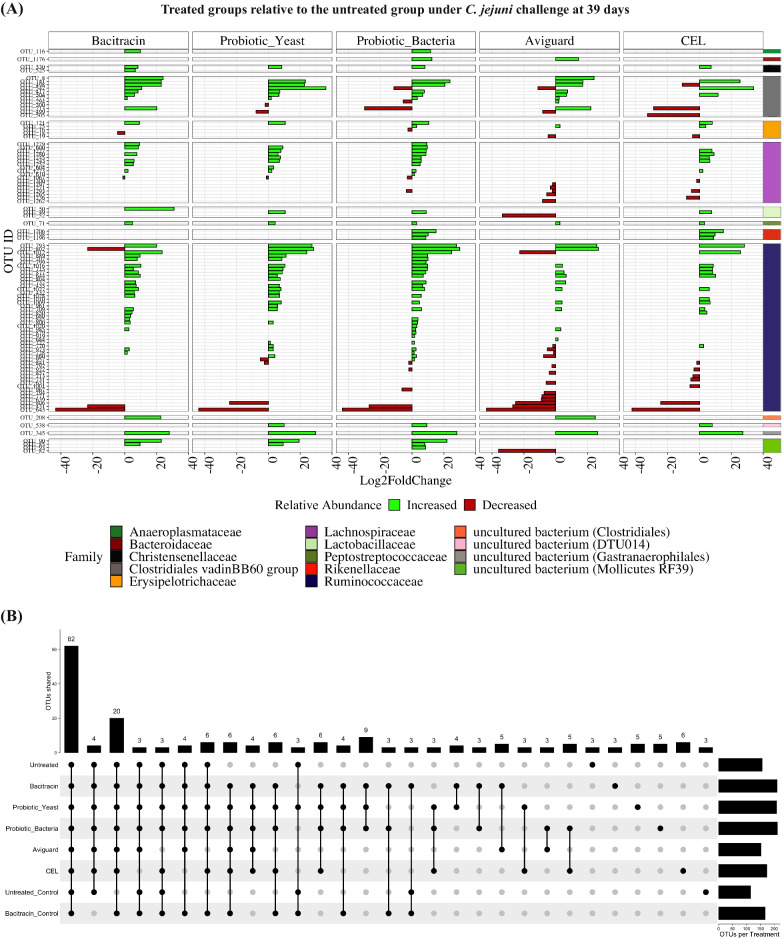
Changes in abundance of OTUs across treatments. **A** Logarithmic fold changes of differential OTUs from differential abundance analyses with DESeq2 at 39 days post-hatch, comparing treated groups to the untreated group under *C. jejuni* challenge. OTUs that are in significantly greater or lesser abundance are represented in green and red coloured bars respectively. OTUs are further grouped by their assigned taxonomic families. Legend lists taxonomic families in order of appearance **B** Vertical bars in the UpSet plot visualizes the number of OTUs unique to each treatment group and the number of OTUs shared between treatment groups at 39 days post-hatch. Horizontal bars represent the total number of OTUs found in each treatment group

Applying DESeq2 [34] to the 39 day samples, 103 OTUs exhibited differential abundance between treated and untreated groups under *C. jejuni* challenge (Fig. 4A). Most were associated with a greater relative abundance in the treated groups. A total of 10 OTUs were consistently increased across all treated groups and OTU643 (assigned to *Ruminococcaceae*) was decreased across all treated groups. OTU19 (*Erysipelotrichaceae*) was found to be decreased only in groups treated with microbial consortia, CEL and Aviguard. Treatment groups that did not significantly lower *C. jejuni* burden relative to the untreated group (bacitracin, *P. acidilactici* and *S. cerevisiae boulardii*) exclusively shared 9 OTUs that were increased and OTU1067 (assigned to *Lachnospiraceae*) was commonly decreased. Treatment groups that resulted in a non-significant increase of *C. jejuni* burden (bacitracin and *P. acidilactici*) shared 5 differential OTUs that were increased. Furthermore, 5 OTUs (3 increased, 2 decreased) were specific to samples from the challenged bacitracin group. A total of 10 OTUs (7 increased, 3 decreased) were specific to *P. acidilactici* while 5 OTUs (3 increased, 2 decreased) were exclusive to *S. cerevisiae boulardii*. Specific to CEL and Aviguard treatment groups, we identified 8 OTUs (1 increased, 7 decreased) and 19 OTUs (2 increased, 17 decreased) respectively.

Overall, there were significant changes in the abundance of OTUs assigned to *Clostridiales vadinBB60 group, Erysipelotrichaceae Lachnospiraceae, Lactobacillaceae, Ruminococcaceae* and *Peptostreptococcaceae* in all samples from challenged birds relative to the challenged untreated birds at both timepoints (Additional file 1: Table S1 and Additional file 2: Table S2; Fig. 4A and Additional file 1: Figure S5A). Of note, all challenged and treated groups at 39 days showed increased relative abundances of a single OTU (OTU71) assigned to *Peptostreptococcaceae*. Further, we noted that OTU116, assigned to *Anaeroplasma,* was consistently found in increased abundance in samples from birds treated with bacitracin or *P. acidilactici*, two of the three treatments that did not mitigate against *C. jejuni* challenge.

Differential OTUs unassigned at the family level but assigned to either *Clostridiales, DTU014 (Clostridia), Gastranaerophilales* and *Mollicutes RF39* were found among samples from treated groups relative to the challenged, untreated group. *Clostridiales* was found at an increased relative abundance in 39-day samples from bacitracin and Aviguard groups. OTU538 assigned to *DTU014 (Clostridia)* was at an increased differential abundance in 39-day samples from birds treated with either *S. cerevisiae boulardii*, *P. acidilactici* or CEL. *Gastranaerophilales* (OTU345) was found at increased relative abundance in all challenged, treatment groups at 39-days. At 39 days, *Mollicutes RF39* were at increased relative abundances among samples from bacitracin, *S. cerevisiae boulardii* and *P. acidilactici* groups and showed decreased relative abundance in Aviguard samples. No differential OTUs assigned to *Mollicutes RF39* were found in CEL samples at 39 days.

Beta diversity analyses and review of taxonomic profiles suggest that each treatment overall resulted in a unique microbial signature. The following sections provide an in-depth review on the key findings associated with each treatment.

### Bacitracin treatment yields signatures of *Akkermansiaceae* in unchallenged conditions

As the impact of AGP-use on *C. jejuni* in poultry is not well known, we compared the effect of bacitracin on the challenged chickens relative to the unchallenged controls [4, 6, 7]. From the alpha diversity studies above, we noted that bacitracin increased the number of bacterial OTUs in the cecal microbiome but did not promote a more evenly distributed community (Table 1; Fig. 2A and Additional file 13: Figure S4A). Beta diversity plots coupled with PERMANOVA and PERMDISP analyses showed that communities treated with bacitracin significantly differed from those associated with other treatments at 30 days and that community composition is affected by *C. jejuni* challenge at both time points (Table 2; Fig. 2B and Additional file 13: Figure S4B). However, we found that 39-day samples taken from challenged birds treated with bacitracin do not differ in community composition from 39-day samples taken from challenged and untreated birds (Table 2; Fig. 2B). This is consistent with the alpha diversity analyses that suggested that the combined effect of *C. jejuni* challenge and bacitracin treatment influenced the cecal microbiome composition. However, both alpha and beta analyses showed that the cecal microbiome was more sensitive to *C. jejuni* challenge than bacitracin treatment.

As noted above, differential abundance testing between bacitracin groups and the untreated controls that were challenged with *C. jejuni* consistently identified an increased relative abundance of the OTU116 (*Anaeroplasma*) (Additional file 1: Table S1 and Additional file 2: Table S2; Fig. 4A and Additional file 14: Figure S5A). While for the day 39 samples, members of *Clostridiales vadinBB60 group* differential at this time point showed only increased relative abundance whereas other challenged, treatment groups exhibited both increased and decreased relative abundance. At 39 days, differential abundance testing identified 50 OTUs, with 45 increased and 5 decreased. On the other hand, differential abundance testing comparing bacitracin to untreated groups in the absence of *C. jejuni* challenge identified increased relative abundances of *Streptococcaceae* and decreased relative abundances in members of *Enterobacteriaceae* among the 39-day samples. Overall, 70 OTUs were identified to be differential over samples taken from untreated and unchallenged birds, where 44 OTUs were increased and 26 OTUs were decreased.

An absence of differential OTUs assigned to *Lactobacillaceae* is noted in 30-day samples taken from challenged birds treated with bacitracin as all other samples taken from challenged birds observed significant changes in OTUs assigned to this family. Reads assigned to *Akkermansiaceae* (*Akkermansia* genus) were notably unique to samples taken from unchallenged birds treated with bacitracin (Fig. 3A and B). Differential abundance testing confirmed *Akkermansiaceae* (*Akkermansia* genus) to be at an increased differential abundance among day 30 samples from unchallenged bacitracin-treated birds over unchallenged and untreated birds (Additional file 3: Table S3).

### Single-strain probiotics have a transient impact on the cecal microbiome during *C. jejuni* challenge

We next focus on the impact of the two single strain probiotics *S. cerevisiae boulardii* and *P. acidilactici*. Alpha diversity analyses suggested that *P. acidilactici* promoted a rich and diverse cecal microbiome (Table 1; Fig. 2A and Additional file 13: Figure S4A). PERMANOVA and PERMDISP analyses indicated that communities at 39 days treated with either single-strain probiotic were similar to those communities obtained from the challenged, untreated birds (Table 2; Fig. 2B). This suggests that over the course of infection by *C. jejuni*, the influence of the probiotics on the communities may have dissipated. Reflecting this possible transient impact, we were unable to associate any reads to *P. acidilactici* in samples taken from *P. acidilactici* treated birds, making it difficult to conclude that the probiotic engrafted under the conditions of this study despite continued administration. Since our sequencing targeted only the 16S rRNA gene, we were unable to monitor the ability of *S. cerevisiae boulardii* to engraft.

Relative to the untreated but challenged controls, differential OTUs found in samples taken from *S. cerevisiae boulardii* treated birds at both time points were not found to be specific to its experimental conditions and were common in other treatment groups (Additional file 1: Table S1 and Additional file 2: Table S2; Fig. 4A and Additional file 14: Figure S5A). A total of 50 OTUs were found differential among the *S. cerevisiae boulardii* group at 39 days, with 43 OTUs increased and 7 OTUs decreased.

The 39-day samples from the *P. acidilactici* group yielded signatures associated with *Bacteroidaceae* and *Rikenellaceae*, absent in the 30-day samples (Fig. 3A, B). Differential abundance testing between samples from the *P. acidilactici* group and samples from the untreated, challenged group confirmed increased differential abundances of *Bacteroidaceae* (*Bacteroides genus;* OTU1176) and *Rikenellaceae* (*Alistipes genus;* OTU1206, OTU1196, OTU1198) at 39 days (Additional file 1: Table S1; Fig. 4A). Along with samples taken from challenged birds treated with bacitracin, samples from the *P. acidilactici* group identified increased relative abundance of OTU116 (assigned to *Anaeroplasma*) across time points (Additional file 1: Table S1 and Additional file 2: Table S2; Fig. 4A and Additional file 14: Figure S5A). Differential abundance testing identified 67 differential OTUs in *P. acidilactici* samples over samples taken from challenged and untreated birds at day 39 where 56 strains are increased, and 11 strains are decreased. Of note, 30-day *P. acidilactici* samples yielded a differential abundance of *Bacillaceae* (OTU46) not found significant in other samples.

### Microbial consortia, Aviguard and CEL promote colonization by *Bacteroidaceae* and *Rikenellaceae* respectively

From the alpha diversity studies above, we noted that treatment with Aviguard resulted in a less complex and less even cecal microbiome than the other challenged groups (Table 1; Fig. 2A and Additional file 13: Figure S4A). Beta diversity analyses further revealed that both Aviguard and CEL promoted distinct microbiome structures (Table 2; Fig. 2B and Additional file 13: Figure S4B).

CEL for example, promoted colonization by *Rikenellaceae*, specifically of the genus *Alistipes*, that was only otherwise observed in 39-day samples taken from birds treated with *P. acidilactici* (Additional file 1: Table S1 and Additional file 1: Table S2; Figs. 3A, B, Fig. 4A and Additional file 14: Figure S5A). From the 44 differential OTUs detected among CEL-treated birds over untreated, challenged birds at 39 days, 30 OTUs are increased and 14 OTUs are decreased. Relative to other treatments, samples from both CEL and Aviguard groups yielded a greater proportion of OTUs assigned to *Ruminococcaceae* that exhibited reduced abundance compared to samples from other treated and challenged groups, potentially reflecting the lower diversity and greater unevenness associated with these samples.

Furthermore, Aviguard promoted a microbiome featuring an abundance of *Bacteroidaceae* (*Bacteroides* genus). Indeed, only samples from Aviguard treated birds and the 39-day samples from birds treated with *P. acidilactici* were found to contain sequence reads assigned to *Bacteroidaceae* (*Bacteroides* genus) (Fig. 3A, B). Aside from *Bacteroidaceae*, samples from the Aviguard group notably observed differential abundances of other taxa that made up its unique microbial signature (Additional file 1: Table S1 and Additional file 2: S2; Fig. 4A and Additional file 14: Figure S5A). The 39-day samples exhibited decreased relative abundances from members of *Lachnospiraceae*, *Lactobacillaceae* and *Mollicutes RF39* that was not observed in other samples at this time point. OTUs assigned to *Christensenellaceae* showed differential abundance among all treatment groups except for samples from the Aviguard group. The decrease or absence of differential taxa mentioned may point to lower diversity and greater unevenness in the microbiome. Furthermore, 39-day samples from birds treated with Aviguard consistently had the least number of differentially abundant strains when compared to samples from other treatments with 24 increased strains and 24 decreased strains. Consistent with alpha diversity analyses, these findings suggested that treatment with Aviguard resulted in a less complex microbial community.

### Chicken cecal microbial communities share a core microbiome embellished with treatment-specific taxa

Our findings from beta diversity analyses, review of taxonomic profiles and differential abundance testing suggest that each treatment resulted in the establishment of a distinct microbial community. To further investigate the uniqueness of each community, we identified OTUs shared between different treatment groups at each time point (Fig. 4B and Additional file 14: Figure S5B). Across all treatments, we identified a core microbiome of 51 and 62 OTUs for the 30-day and 39-day samples respectively. This increase might be associated with an increase in microbial complexity as the birds age [35, 36]. Also notable is a set of nine taxa common to all 30-day samples with the exception of those samples collected from untreated and unchallenged birds. These increased to 20 taxa for the 39-day samples. This again highlights the effect of *C. jejuni* challenge and dietary additives on the cecal microbial community.

At the 30-day timepoint, samples from challenged birds that either received bacitracin or *P. acidilactici* shared 12 OTUs that were not found in any other sample, including members of *Mollicutes RF39*, *Anaeroplasmataceae*, *Erysipelotrichaceae*, *Ruminococcaceae*, *Christensenellaceae* and *Lachnospiraceae*. However, this dropped to just three OTUs at 39 days, which were assigned to *Anaeroplasmataceae* and *Ruminococcaceae*. Coincidentally, bacitracin and *P. acidilactici* were treatments that failed to lower CFUs of *C. jejuni*. This suggests that failure to alleviate *C. jejuni* burden may prevent the former OTUs from persisting within the ceca. To investigate further, we calculated Spearman’s rank correlation coefficients for each of the 12 OTUs against *C. jejuni* sequence counts at 30 days. Only one OTU, assigned to *Lachnospiraceae,* was significantly (negatively) correlated with *C. jejuni* sequence counts (R = −0.89, *p* = 0.033; Spearman’s rank correlation coefficient).

CEL was notable in having the highest number of exclusive OTUs (13 and 6 OTUs at day 30 and 39 respectively), potentially representing components of the CEL consortium itself. For the 30-day samples, these included members of *Ruminococcaceae*, *Clostridiales vadinBB60 group*, *Lachnospiraceae*, *Peptococcaceae* and *Rikenellaceae*, while for the 39-day samples, these included members of *Christensenellaceae*, *Peptococcaceae*, *Ruminococcaceae* and *Lachnospiraceae*. This highlights the unique ability of CEL to promote the persistence of taxa not associated with any other treatment.

## Discussion

Foodborne illness in humans caused by *C. jejuni* through contaminated meat products has directed efforts to reduce avian colonization [37]. In the present study, we assessed and compared the efficacy of four industrially relevant microbial interventions (Aviguard, CEL, *S. cerevisiae boulardii* and *P. acidilactici*) and bacitracin to reduce *C. jejuni* burden. We further explored the impact of each treatment on the composition of the cecal microbiota. Here we focused on the cecum as an important site for *C. jejuni* colonization and the role of the cecal microbiota on host immunity and restricting colonization by pathogens [38]. Moreover, the cecum plays a key role in nutrient absorption and the production of short chain fatty acids [38]. Examining the impact of different treatments under standardized experimental conditions (housing, genotype, geography, time points and diet) allows the identification of meaningful differences in terms of taxonomic composition.

Bacitracin consists of high molecular weight polypeptides with demonstrated antimicrobial activity against Gram-positive microorganisms by interfering with the formation of the bacterial cell wall [1]. Administered at sub-therapeutic quantities, bacitracin promotes a healthy gut microbiome, resulting in enhanced growth and preventing infections involving food safety pathogens, as well as those impacting flock health [3–5, 8]. For example, dietary supplementation with bacitracin can reduce *C. perfringens* count in the chicken microbiota and prevent necrotic enteritis [1, 6]. However, bacitracin’s impact on *C. jejuni*, a Gram-negative bacterium that causes campylobacteriosis, is not well defined [4, 6, 7, 29, 39, 40]. Consistent with reports of an absence of appropriate targets and/or low affinity of binding of bacitracin [41–43], we found that treatment with bacitracin did not lower *C. jejuni* burden. Further, through the preferential targeting of Gram-positive bacteria, bacitracin treatment may have opened a niche in the gut environment and allowed greater *C. jejuni* colonization. In addition, it is not recommended to reduce *C. jejuni* burden in poultry with AGP administration since birds are largely asymptomatic and *C. jejuni* has been found to readily acquire antibiotic resistance mechanisms [43]. For example, use of fluoroquinolones as an AGP has been linked to the development of ciprofloxacin-resistant *Campylobacter* in humans [37]. Along with previous literature, results from this study have highlighted that AGP use in poultry production is not ideal and calls to the need for alternatives.

Outside of its ability to mitigate *C. jejuni* infection, in the absence of this pathogen, we found bacitracin treatment promoted colonization of the ceca by *Akkermansia*. Although we were unable to define the *Akkermansia* OTU to the species level, it has been suggested that *Akkermansia muciniphila* may improve host metabolic functions and immune responses [44]. *A. muciniphila* is an intestinal symbiont that colonizes the outer mucosal layer, an anaerobe that degrades the mucin for carbon and nitrogen elements and other beneficial by-products that also exerts competitive inhibition on other pathogenic bacteria that degrade mucin [44]. Current evidence shows that *A. muciniphila* is a promising therapeutic target in microbiome-related diseases such as colitis, metabolic syndrome, immune diseases and cancer [44] and may therefore represent one mechanism by which bacitracin exerts a positive influence on gut health. Interestingly, several studies have reported antibiotic treatment promotes *A. muciniphila* to be the most abundant member of the gut microbiome [45–47]. For example, vancomycin treatment in mice reduced the abundance of *Firmicutes and Bacteroides*, while promoting *A. muciniphila*, and was associated with reducing cumulative diabetes incidence [46]. A separate study of two human patients unexpectedly identified a high proportion (> 40% of all taxa) of *A. muciniphila* in the gut microbiota after receiving broad-spectrum antibiotic treatments [47].

Supplementation with *S. cerevisiae boulardii* has been reported to reduce *C. jejuni* abundance in fecal samples, lead to a higher abundance of beneficial microorganisms and positively influence intestinal mucosa architecture in broiler chickens, with an overall improvement on growth performance [48]. While we did note a slight reduction in *C. jejuni* burden due to treatment with *S. cerevisiae boulardii* relative to the bacitracin-treated controls, this decrease was not statistically significant. Sample size may need to be increased in follow-up studies to determine that *S. cerevisiae boulardii* is effective in reducing *C. jejuni* burden relative to bacitracin treatment. The aforementioned study also reported higher abundances of *Lactobacillaceae* following *S. cerevisiae boulardii* treatment, which reflects other reports of this family to be negatively correlated with *C. jejuni* [48–51]. Although we found *Lactobacillaceae* groups to be differentially abundant among communities treated with *S. cerevisiae boulardii* in this study, it was not specific to this treatment. Further, samples from birds treated with *S. cerevisiae boulardii* in our study did not indicate colonization of other bacteria unique to this treatment. Similar to our findings, the prior study reported no significant difference in alpha and beta diversity between control samples and treated samples [48]. The surface of *S. cerevisiae boulardii* has also been found to directly bind to *Salmonella* potentially preventing invasion in the host and lowering its colonization in the poultry gut [49, 52, 53]. Based on results from this study and previous reports, *S. cerevisiae boulardii* is a potentially promising alternative to bacitracin for foodborne pathogens of concern in poultry production.

The other single strain probiotic used in this study, *P. acidilactici,* has been shown to significantly decrease counts of *Campylobacter* in free-range finishing pigs before slaughter [54]. Moreover, a small cocktail of probiotics that included *P. acidilactici* isolated from a healthy chicken gut significantly inhibited *C. jejuni* growth in broiler chickens [39]. Another study in broiler chickens reported a significant reduction of *C. jejuni* burden after administration of probiotic preparations that included *S. cerevisiae boulardii* and *P. acidilactici* [55]. In contrast, we did not see any reduction of pathogen burden through *P. acidilactici* supplementation, relative to treatment with bacitracin. One explanation for this inconsistency with previous reports of lowered burden of *C. jejuni* may be the limited ability of *P. acidilactici* to colonize the birds in our study, as indicated by the inability of 16S rDNA surveys to detect *P. acidilactici* among samples. At the same time, it should be appreciated that these surveys rely on measures of *relative abundance* rather than *absolute abundance* and that other abundant taxa may simply be masking the presence of *P. acidilactici*. Hence an alternative explanation may reflect *P. acidilactici*’s ability to secrete pediocins, antimicrobial peptides that target and inhibit the growth of gram-positive bacteria [56]. Thus, *P. acidilactici* may be acting indirectly on *C. jejuni* through removing taxa that may otherwise restrict the growth of the gram-negative pathogen. This positive influence on *C. jejuni* might counterbalance any direct impact of *P. acidilactici* on the pathogen itself. Beyond any impact on *C. jejuni*, treatment with *P. acidilactici* was associated with changes in the relative abundance of other taxa, notably a single OTU assigned to *Bacillus*, a commonly used single-strain probiotic used in livestock, reported to produce bacteriocins and antimicrobial peptides [5, 12]. We also identified a single OTU assigned to *Bacteroidaceae* and several OTUs assigned to *Rikenellaceae*, illustrating the distinct impact of this probiotic on the cecal microbiome.

Aviguard is a competitive exclusion (CE) product administered to newly hatched chicks to promote the formation of a stable and diverse microbiome that exerts colonization resistance. It is a partially characterized, freeze-dried mixture of live commensal bacteria derived from the gut microbiota of specific-pathogen-free (SPF) adult chickens [57]. In our study, Aviguard supplementation mirrored the success found in other studies where it was able to inhibit intestinal colonization of other enteric pathogens such as *Clostridium perfringens* and *Salmonella typhimurium* in chickens [29–32]. Aviguard treatment was associated with reduced alpha diversity and reduction in the number of differentially abundant taxa. This suggests that Aviguard, which is cultured from intestinal samples obtained from healthy adult birds, may have rapidly engrafted in the ceca of day old chicks, establishing a microbiome of reduced complexity, potentially dominated by a few highly abundant taxa [32]. This results in a distinct microbiome (as evidenced through beta diversity analyses) and a unique ability to promote colonization by *Bacteroidaceae*.

Despite also representing a complex microbial consortium, CEL did not elicit the same effect on alpha and beta diversity as Aviguard. However, it was notable that beta diversity calculations of challenged 39-day samples from bacitracin, *S. cerevisiae* boulardii, P*. acidilactici* and untreated groups did not have a significant difference from each other. This suggests that microbial consortia treatments result in very distinct microbiomes. This is further reflected by differential abundance of members of *Rikenellaceae* being associated only in CEL groups, as well as 39-day samples from the *P. acidilactici* group.

Both *Bacteroidaceae* and *Rikenellaceae* belong to the *Bacteroidetes* phylum, which includes bacteria considered to be a stable part of the GI microbiota and are major producers of short-chain fatty acids (SCFAs), key molecules involved in host homeostasis and disease state [58–60]. Further studies, perhaps through metabolomics, are required to determine the impact of shifts of diversity on the production of SCFAs by the high relative abundance of *Bacteroidaceae* and *Rikenellaceae* in the cecal microbiome under Aviguard and CEL treatment respectively. *Rikenellaceae* (*Alistipes* genus) is implied to play a critical role in inflammation and disease but its exact mechanisms in the microbiome are yet to be completely elucidated [58, 59, 61]. The observed differential abundance of *Rikenellaceae* was consistent with previous reports of this family being sensitive to fluctuations in the gut microbiome caused by supplementation with antibiotics or probiotics [62, 63].

Differential abundances of *Lactobacillaceae* (*Lactobacillus* genus) were found among samples taken from challenged birds. Members from the *Lactobacillaceae* are widely used as probiotics to help recover a healthy microbial community after dysbiosis [49, 64–66] and are known to be successful in reducing enteric diseases and maintaining a healthy microbiota in poultry [13]. *Lactobacillus* strains can lower pathogen burden by decreasing the gut pH via lactic acid secretion and are known to reduce the incidence of *Salmonella* spp., *Clostridium perfringens* and *Campylobacter* infections [48, 67, 68]. Moreover, other studies have associated decreases in *Lactobacillaceae* populations with increased *Campylobacter* abundance or stress [12, 48, 50, 51]. Although strains of *Lactobacillus* have been suggested as probiotics that can mitigate against colonization by pathogens, we found no association between differential abundance of OTUs assigned to this genus with *C. jejuni* burden in the present study.

Interestingly, higher alpha diversity measures were observed in challenged groups and samples from either bacitracin or *P. acidilactici* groups while lower alpha diversity measures were seen in the Aviguard group. Coincidentally, treatments of bacitracin and *P. acidilactici* failed to reduce *C. jejuni* burden compared to other challenged groups while Aviguard treatment succeeded. In contrast to our findings, previous studies have reported that *C. jejuni* does not affect the alpha diversity in the cecal microbiota [69–71]. Also in contrast to our study’s findings, bacitracin has also been reported to decrease alpha diversity [72]. Traditionally, increased diversity in the microbiome is considered beneficial to host health while decreased diversity points to a decline [73, 74]. The current study presents contrasting results where microbiomes with lower pathogen burden observe lower diversity, highlighting a need to broaden our understanding of diversity scores to better define the microbial community’s role in host health.

Our data shows that the *Firmicutes* phylum is predominant in the chicken cecal microbiome, largely consisting of *Ruminococcaceae*, *Lachnospiraceae* and *Clostridiales vadin BB60 group*, with *Christensenellaceae*, *Peptostreptococcaceae* and *Erysipelotrichaceae* at lower percentages. Indeed, bacteria belonging to the phylum, *Firmicutes*, are the dominant taxa in older birds [35, 38]. *Ruminococcaceae* and *Lachnospiraceae* are major SCFA-producing bacterial members of the gut microbiome [38, 49, 75]. *Ruminococcaceae* and *Lachnospiraceae* are both obligate anaerobes that metabolise energy by anaerobic respiration and fermentation and are major producers of fermentation products such as the SCFAs: butyrate, acetate, lactate and formate [49, 75]. The *Clostridiales vadin BB60 group* is largely unclassified and uncharacterized in its role in metabolism and in the microbiome whereas *Christensenellaceae* has been linked to lower BMI and overall gut health in humans [38]. Samples in this study were taken near the age of slaughter and yield relative abundances of *Tenericutes* groups such as *Mollicutes RF39* and *Anaeroplasmataceae* (*Anaeroplasma*), consistent with other studies investigating the mature chicken cecal microbiome [38, 51]. Members of the phylum, *Anaeroplasma* have also been reported to increase in cecal microbiome of heat-stressed broilers [76]. Interestingly, we noted the increased relative abundance of *Anaeroplasma* in samples taken from birds treated with bacitracin or *P. acidilactici*, treatments that did not mitigate against *C. jejuni* challenge. We therefore speculate that the increased abundance in *Anaeroplasma* may have resulted from the inability of the bacitracin and *P. acidilactici* treatments to reduce stressors to the chicken ceca. Moreover, *Gastranaerophilales* was also seen at differential abundances among samples in this study. Although the role of *Gastranaerophilales* in the microbiome is uncharacterized, this group is thought to aid host digestion by fermentation and are a source of vitamins B and K [77]. Overall, the present study showed that the core chicken microbiome is dominated by *Firmicutes* in older birds with other phyla such as *Bacteroidetes, Tenericutes, Cyanobacteria* and *Verrucomicrobia* in lower proportions. This reflects previous studies that have shown that *Firmicutes, Bacterioidetes*, and *Proteobacteria* make up the core cecal microbiota of birds, with *Firmicutes* dominating in older birds [9, 35, 78, 79].

## Conclusions

Given the varying effects of the microbial interventions, with each conferring a specific benefit (e.g. colonization by *Bacteroidetes* or *Rikenellaceae*), our findings suggest that the application of each probiotic may depend on the individual requirements of the farm and/or flock receiving the additives. Overall, microbial-based products are a promising alternative to AGPs addressing the problem of rising antibiotic resistance mechanisms and reducing colonization by *C. jejuni* in poultry to help mitigate the incidence of human campylobacteriosis.

## Methods

### Animal trials

All experimental procedures were approved by the University of Guelph Animal Care Committee and conducted according to specifications of the Canadian Council on Animal Care. One hundred and sixty broiler (Ross 708, Aviagen Inc. Huntsville, AL) chickens were divided randomly into 8 groups and assigned to one of 6 treatments at day one of age. On day one, group 1 and 2 received complex microbial consortia Aviguard and CEL respectively in their drinking water as one shot, for less than 24 h. Group 3 received probiotic bacteria (*Pediococcus acidilactici CNCM I-4622*), group 4 received probiotic yeast (*Saccharomyces cerevisiae boulardii CNCMI-1079*), and group 5 and 6 received AGP (bacitracin). Group 3, 4, 5 and 6 received their supplements in their feed throughout the entire experimental period. Group 7 and 8 did not receive any dietary additives and were fed a normal diet.

Prior to administration, each complex consortium sachet was dissolved in 500 mL deionized water and provided with 1 L of deionized water to avoid the effects of chlorine. *P. acidilactici* and *S. cerevisiae boulardii* supplementation was added to 150 kg of standard feed according to commercial guidelines, resulting in a dose of 1 × 10^9^ CFU/kg of feed for each product. All probiotics were sourced from Lallemand SAS, Blagnac, France. An amount of 110 g of AGP (bacitracin) was added to 220 kg of standard feed for AGP treatment groups.

On day 14, all groups, except for groups 6 and 8 received an oral gavage of 10^7^ CFUs of *C. jejuni* (strain 81–176) in 500 µl PBS. Fecal droppings were collected every 5 days (starting from day 1 post-hatch to day 39 post-hatch). Approximately 0.5–1 g of cecal contents were collected at day 30 and 39 of age (n = 10 birds per time point and treatment group, total of 158 samples for sequencing). After sample collection, tenfold serial dilutions in PBS were plated onto Muller Hinton agar containing Preston *Campylobacter* Selective Supplement, an antibiotic cocktail that prevents growth of other bacteria. Plates were incubated in microaerophilic conditions at 41 °C and CFUs of *C. jejuni* were enumerated after 40–48 h and expressed as log_10_ *Campylobacter* /g of cecal content.

### Next-generation sequencing (NGS) and sequence processing

Chicken cecal samples were submitted to the Integrated Microbiome Resource at Dalhousie University, Nova Scotia, Canada for 16S rRNA gene high-throughput Illumina sequencing. Microbial communities were analyzed through the V4/V5 hypervariable region of the 16S rRNA gene to capture bacterial taxonomic groups (Primer sequences: 515f (5’-GTGYCAGCMGCCGCGGTAA-3’) and 926r (5’-CCGYCAATTYMTTTRAGTTT-3’)) [80]. The initial quality filtering steps were performed on demultiplexed paired-end reads using the QIIME2 platform [81]. Primers were trimmed using the Cutadapt plugin within QIIME2 [82]. Reads were denoised, dereplicated, filtered for chimeras and merged using the DADA2 plugin within QIIME2 [83]. Sequences were clustered into Operational Taxonomic Units (OTUs) according to a similarity set at 97% based on a de novo assignment [84]. Taxonomic assignment was performed against the SILVA 132 database using the Scikit-learn plugin within QIIME2 at a confidence threshold of 70%, and a phylogenetic tree was produced [85–87].

### Statistical analyses

Downstream analyses and graphical outputs were generated in R v.3.6.2 with a combination of R packages including Phyloseq v.1.30.0, Vegan v.2.5.6, PairwiseAdonis v.0.0.1, DESeq2 v.1.26.0 and ComplexHeatmap v. 2.5.3 [34, 88–92]. The Phyloseq package was used to perform normalization, visualize abundance of microbial taxonomic composition and estimate both alpha and beta diversity metrics. *P* < 0.05 was considered significant for all statistical tests. The effects of microbial-based treatments on colony counts (CFUs) relative to control groups was analyzed with non-parametric Kruskal–Wallis tests, followed by pairwise comparisons with Dunn’s test adjusted with the Benjamini–Hochberg (B–H) method.

Reads were normalized by rarefying to even sequencing depths before calculating species richness (Observed amplicon sequence variants (ASVs) and Chao1) and evenness (Shannon and Simpson index) for alpha diversity estimations. A minimum sequence depth of 5753 was kept and taxa not seen more than 5 times in at least 5 samples were removed. Non-parametric Kruskal–Wallis tests were conducted, followed by subsequent pairwise comparisons with Wilcoxon rank sum test adjusted with the Benjamini–Hochberg (B–H) method to compare alpha diversity metrics among groups.

Total sum scaling was used to normalize raw counts prior to beta diversity analyses and visualizing taxonomic composition. For beta diversity analyses, dissimilarity matrices between samples were calculated with the Bray Curtis method and was visualized with Non-Metric Multidimensional Scaling (nMDS) ordination technique. The nMDS plots in this study were visualized on two dimensions as it exhibited the most separation between treatment groups as opposed to four dimensions. nMDS calculations do not maximize the variability associated with individual axes of the ordination unlike eigenvector-based methods such as principal component analysis, principal coordinates analysis or correspondence analysis [93]. Permutational multivariate analysis of variance (PERMANOVA) with the adonis function from R’s Vegan package was conducted to evaluate the significance of separation between centroids of samples grouped by treatment or time point but is sensitive to multivariate dispersion. Conducted in parallel to PERMANOVA, permutation multivariate analysis of dispersion (PERMDISP) was computed with the function betadisper and permutest from the Vegan package to assess if the distribution or spread of two sample groups are significantly different. Pairwise PERMANOVA and PERMDISP tests were also calculated and adjusted with B–H method.

Differential abundance tests of raw counts were generated with DESeq2 to identify differential taxa among samples. Normalized counts were extracted from DESeq2 to conduct Spearman’s rank tests. Spearman’s rank test was conducted on challenged samples to find taxa negatively/positively correlated with *C. jejuni* and to compare sequence counts to CFUs of *C. jejuni*. UpSet plots were created to visualize OTUs shared between or unique among treatment groups, which was implemented with ComplexHeatmap. Prior to building UpSet plots, a filtering step was applied to raw counts where taxa not seen more than 5 times in at least 5 samples were removed.

## Supplementary Information

**Additional file 1**. **Table S1.** Results from differential abundance analyses with DESeq2 of 39-day samples, comparing treated groups to the untreated group under *C. jejuni* challenge.

**Additional file 2**. **Table S2.** Results from differential abundance analyses with DESeq2 of 30-day samples, comparing treated groups to the untreated group under *C. jejuni* challenge.

**Additional file 3**. **Table S3.** Results from differential abundance analyses with DESeq2, comparing the bacitracin group to the untreated group in the absence of *C. jejuni* challenge.

**Additional file 4**. **Table S4.** Results from differential abundance analyses with DESeq2, comparing samples at 39 days to samples at 30 days post-hatch within each of the 8 treatment groups.

**Additional file 5**. **Table S5.** Results from Kruskal–Wallis rank sum tests comparing the alpha diversity indices (Observed, Chao1, Shannon, Simpson) of samples grouped according to treatment at each time point.

**Additional file 6**. **Table S6.** P-values from post-hoc pairwise Wilcoxon rank sum tests comparing the alpha diversity indices (Observed, Chao1, Shannon, Simpson) of samples grouped according to treatment at each time point.

**Additional file 7**. **Table S7.** P-values from Wilcoxon rank sum tests comparing the alpha diversity indices (Observed, Chao1, Shannon, Simpson) of samples grouped according to days post-hatch within each treatment group.

**Additional file 8**. **Table S8.** Influence of treatment on microbiome structure at each time point. Results from PERMANOVA and PERMDISP analyses of the Bray–Curtis distances conducted in parallel.

**Additional file 9**. **Table S9.** Significance of separation between centroids of samples grouped by treatment. Results from pairwise PERMANOVA analyses of the Bray–Curtis distances.

**Additional file 10**. **Table S10.** Significance of the distribution of samples grouped by treatment. Results from pairwise PERMDISP analyses of the Bray–Curtis distances. Conducted in parallel to pairwise PERMANOVA analyses to place confidence in a significant result.

**Additional file 11**. **Table S11.** Influence of days post-hatch on microbiome structure within each treatment group. Results from PERMANOVA and PERMDISP analyses of the Bray–Curtis distances conducted in parallel.

**Additional file 12**. **Figure S1.** Samples collection yielded 158 cecal content samples, which were sent for 16S rRNA sequencing. **(A)** After quality filtering, 2,195,510 paired-end reads were retained with a median of 13,562 reads (5753–29,542 reads) per sample. (**B)** Sequences were clustered into 1305 Operational Taxonomic Units (OTUs) based on a de novo assignment with similarity set at 97%. **Figure S2.** Scatter plots showing the Spearman correlation between enumerated CFUs of *C. jejuni* and sequence counts assigned to *C. jejuni* in samples at **(A)** 30 and **(B)** 39 days post-hatch

**Additional file 13**. **Figure S3.** Bar plots representing the relative abundance of *C. jejuni* in all samples, grouped by treatment and age. **Figure S4.** Alpha and beta diversity of cecal microbial communities. **(A)** Boxplots representing alpha diversity metrics of richness (observed number of OTUs and Chao1) and evenness (Shannon and Simpson) for 30-day samples grouped according to treatment (no. of OTUs at 97% similarity). Each point represents the diversity score for a sample, colour-coded according to treatment. **(B)** Non-metric multidimensional scaling (nMDS) plot based on Bray–Curtis dissimilarity matrix on relative abundance data in 30-day samples. Colours indicate treatment group

**Additional file 14**. **Figure S5. (A)** Logarithmic fold changes of differential OTUs from differential abundance analyses with DESeq2 at 30 days post-hatch, comparing treated groups to the untreated group under *C. jejuni* challenge. OTUs that are in significantly greater or lesser abundance are represented in green and red coloured bars respectively. OTUs are further grouped by their assigned taxonomic families. Legend lists taxonomic families in order of appearance. **(B)** Vertical bars in the UpSet plot visualizes the number of OTUs unique to each treatment group and the number of OTUs shared between treatment groups at 30 days post-hatch. Horizontal bars represent the total number of OTUs found in each treatment group

## Data Availability

The sequence data generated during the current study are available from the NCBI Sequence Read Archive with the BioProject identifier PRJNA767870.

## Abbreviations

AGP: Antibiotic growth promoter
ASV: amplicon sequence variant
B–H: Benjamini–Hochberg
CE: Competitive exclusion
CFU: Colony forming unit
FMT: Fecal microbiota transplantation
GI: Gastro-intestinal
NE: Necrotizing enteritis
nMDS: Non-metric multidimensional scaling
OTU: Operational taxonomic unit
PERMANOVA: Permutational multivariate analysis of variance
PERMDISP: Permutational analysis of multivariate dispersions
SCFA: Short-chain fatty acid
SPF: Specific-pathogen-free
