# Supplementary figures and images for: Performance of distinct microbial based solutions in a Campylobacter infection challenge model in poultry
Source: Anim Microbiome. 2022 Jan 3;4:2. doi: 10.1186/s42523-021-00157-6 (PMC8722297; doi:10.1186/s42523-021-00157-6)

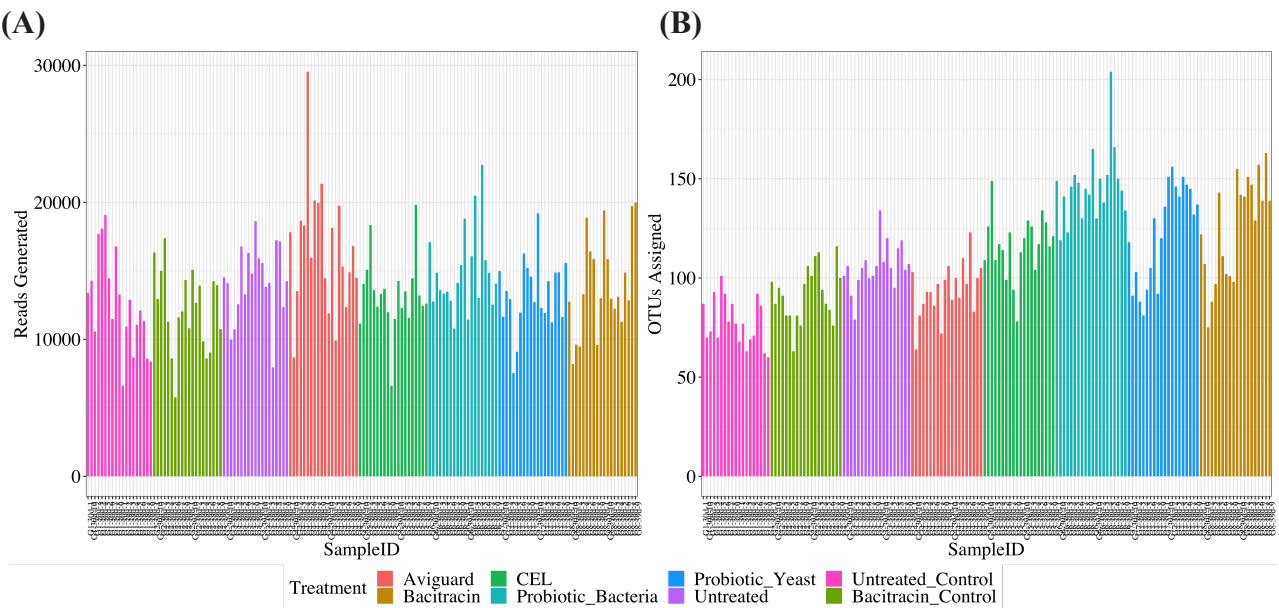

**Supplemental Figure 1**

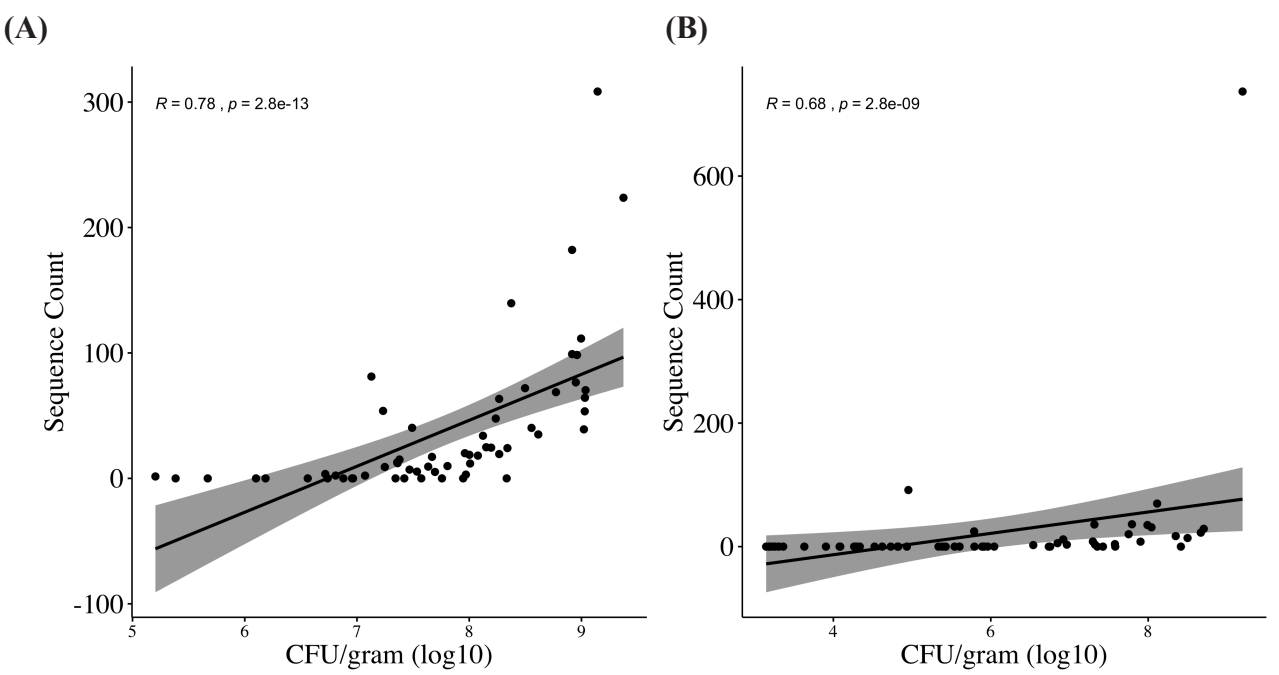

**Supplemental Figure 2**

Supplement: Supplementary file 12 — Additional file 12. Figure S1. Samples collection yielded 158 cecal content samples, which were sent for 16S rRNA sequencing. (A) After quality filtering, 2,195,510 paired-end reads were retained with a median of 13,562 reads (5753–29,542 reads) per sample. (B) Sequences were clustered into 1305 Operational Taxonomic Units (OTUs) based on a de novo assignment with similarity set at 97%. Figure S2. Scatter plots showing the Spearman correlation between enumerated CFUs of C. jejuni and sequence counts assigned to C. jejuni in samples at (A) 30 and (B) 39 days post-hatch [file 42523_2021_157_MOESM12_ESM.pdf]

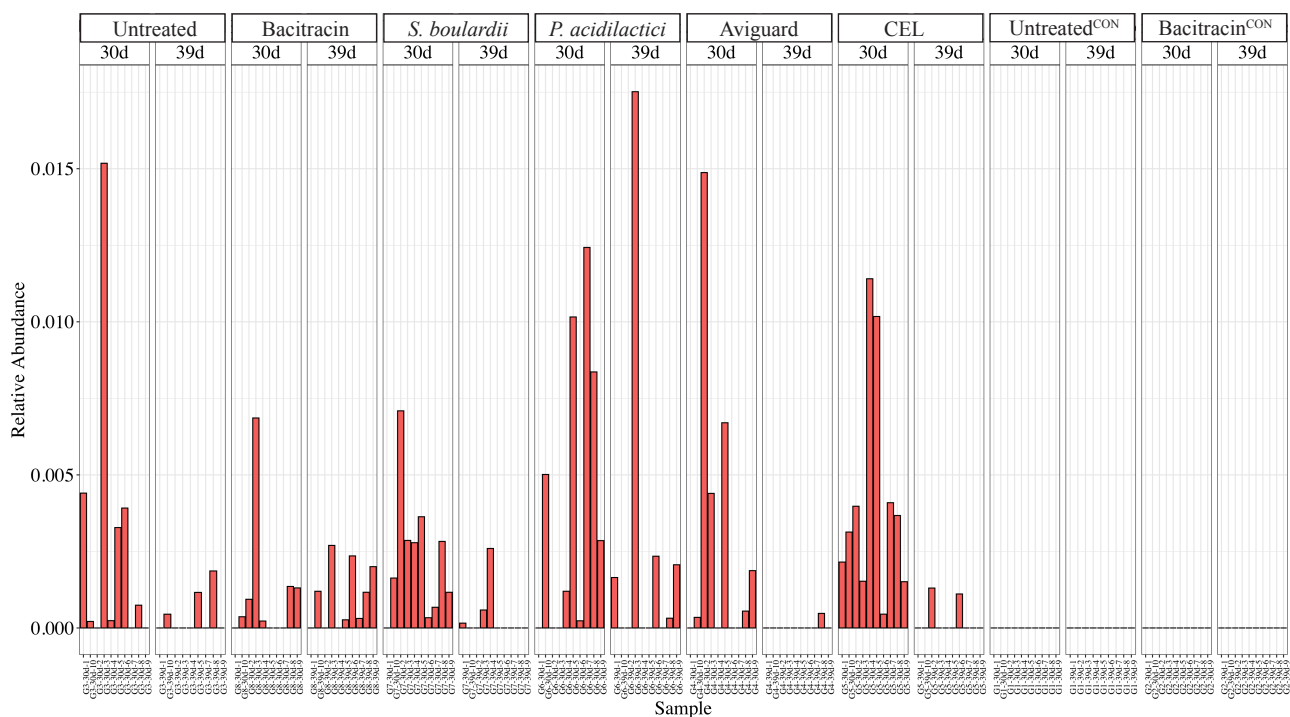

**Supplemental Figure 3**

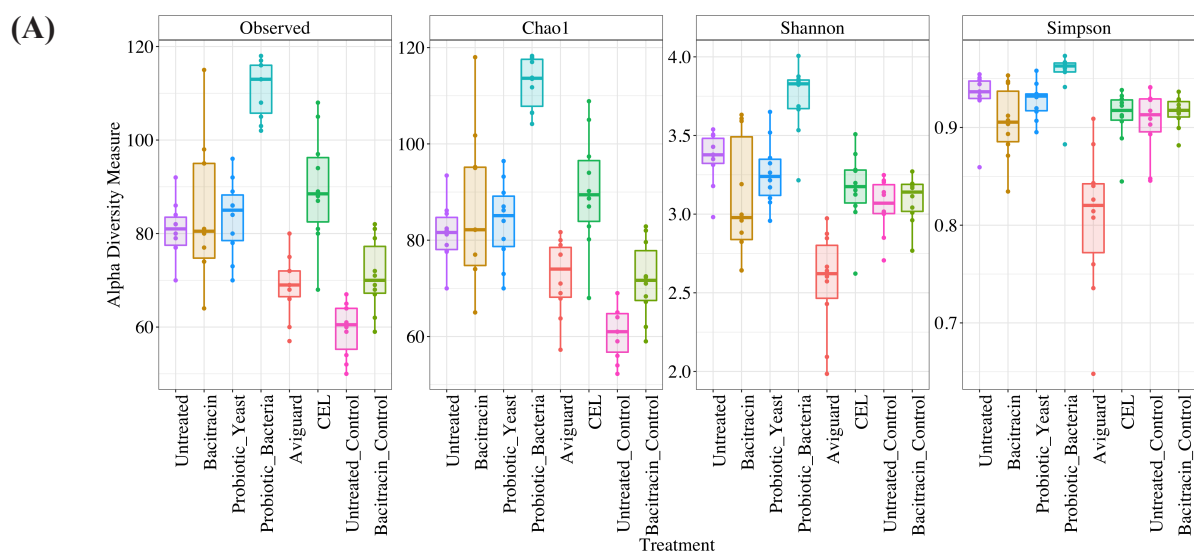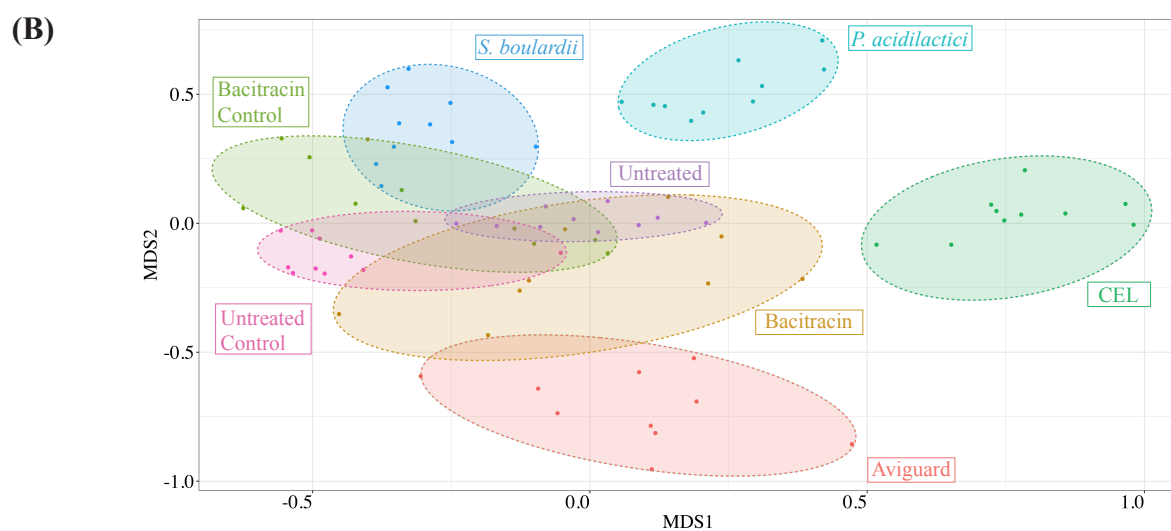

**Supplemental Figure 4**

Supplement: Supplementary file 13 — Additional file 13. Figure S3. Bar plots representing the relative abundance of C. jejuni in all samples, grouped by treatment and age. Figure S4. Alpha and beta diversity of cecal microbial communities. (A) Boxplots representing alpha diversity metrics of richness (observed number of OTUs and Chao1) and evenness (Shannon and Simpson) for 30-day samples grouped according to treatment (no. of OTUs at 97% similarity). Each point represents the diversity score for a sample, colour-coded according to treatment. (B) Non-metric multidimensional scaling (nMDS) plot based on Bray–Curtis dissimilarity matrix on relative abundance data in 30-day samples. Colours indicate treatment group [file 42523_2021_157_MOESM13_ESM.pdf]
