# Supplementary material for: Performance of distinct microbial based solutions in a Campylobacter infection challenge model in poultry
Source: Anim Microbiome. 2022 Jan 3;4:2. doi: 10.1186/s42523-021-00157-6 (PMC8722297; doi:10.1186/s42523-021-00157-6)
Supplement: Supplementary file 14 — Additional file 14. Figure S5. (A) Logarithmic fold changes of differential OTUs from differential abundance analyses with DESeq2 at 30 days post-hatch, comparing treated groups to the untreated group under C. jejuni challenge. OTUs that are in significantly greater or lesser abundance are represented in green and red coloured bars respectively. OTUs are further grouped by their assigned taxonomic families. Legend lists taxonomic families in order of appearance. (B) Vertical bars in the UpSet plot visualizes the number of OTUs unique to each treatment group and the number of OTUs shared between treatment groups at 30 days post-hatch. Horizontal bars represent the total number of OTUs found in each treatment group [file 42523_2021_157_MOESM14_ESM.pdf]

(A) Treated groups relative to the untreated group under *C. jejuni* challenge at 30 days

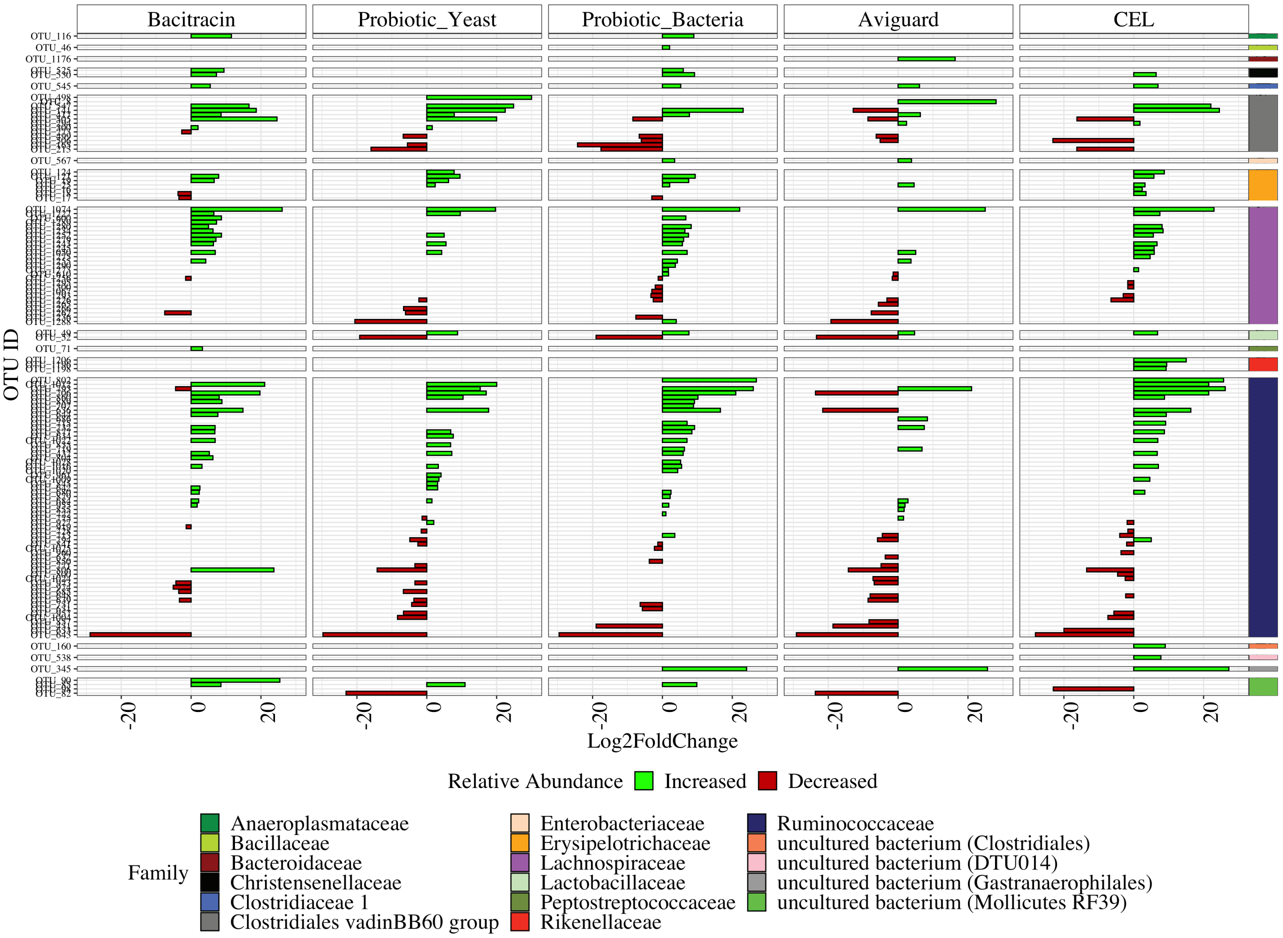

(B)

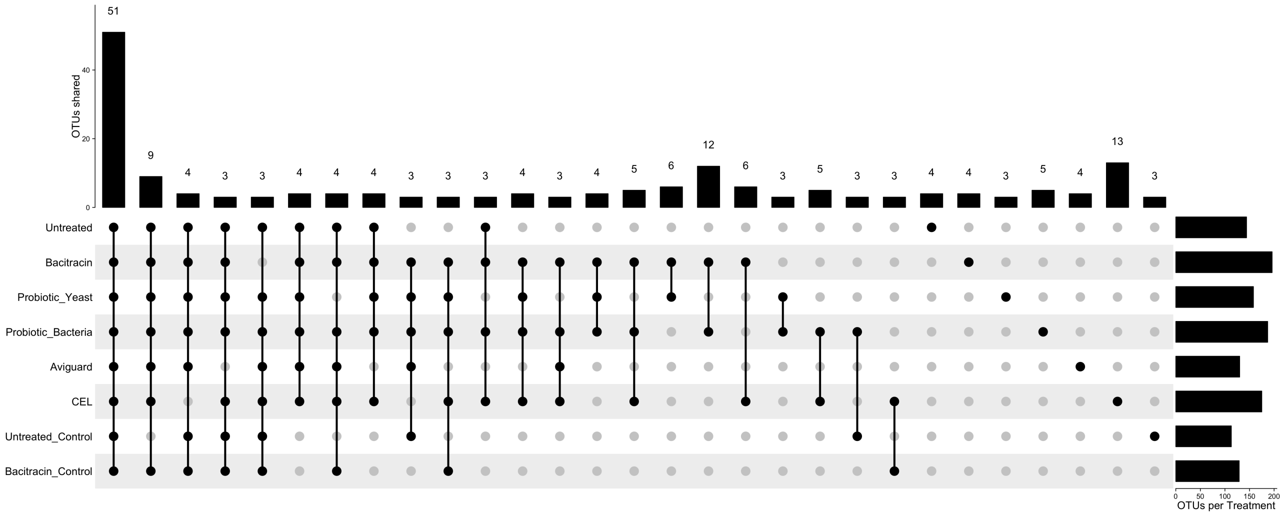

Supplemental Figure 5
